# Advancements in Cellulose for Eco-Friendly Lubricant Applications: A Review on Tribological Properties

**DOI:** 10.1021/acsomega.5c05037

**Published:** 2025-08-14

**Authors:** Dieter Rahmadiawan, Shih-Chen Shi, Navid Aslfattahi, Anna Niska Fauza, Zahrul Fuadi

**Affiliations:** † Department of Mechanical Engineering, 34912 National Cheng Kung University (NCKU), Tainan 70101, Taiwan; ‡ Department of Mechanical Engineering, 175470 Universitas Negeri Padang, 25173 Padang, Sumatera Barat, Indonesia; § Department of Fluid Dynamics and Thermodynamics, Faculty of Mechanical Engineering, 48220 Czech Technical University in Prague, Prague 166 07, Czech Republic; ∥ IINM, Warwick Manufacturing Group, University of Warwick, Coventry CV4 7AL, U.K.; ⊥ Department of Mechanical and Industrial Engineering, 175503 Universitas Syiah Kuala, Banda Aceh 23111, Indonesia

## Abstract

Cellulose, a sustainable and biodegradable biopolymer, has emerged as a promising candidate for lubricant additives due to its ability to form protective boundary layers, reduce surface roughness, and enhance load-bearing capacity. This review explores the underlying tribological mechanisms, such as the mending effect, physical adsorption, and hydrogen bonding, which contribute to the performance of cellulose-based lubricants. Various applications are then discussed across liquid, semisolid, and solid lubrication systems. Notably, cellulose nanocrystals (CNCs) and hydroxyethyl cellulose (HEC) demonstrate strong friction and wear reducing performance. The paper also identifies some research gaps and limitations that need to be addressed.

## Graphical abstract

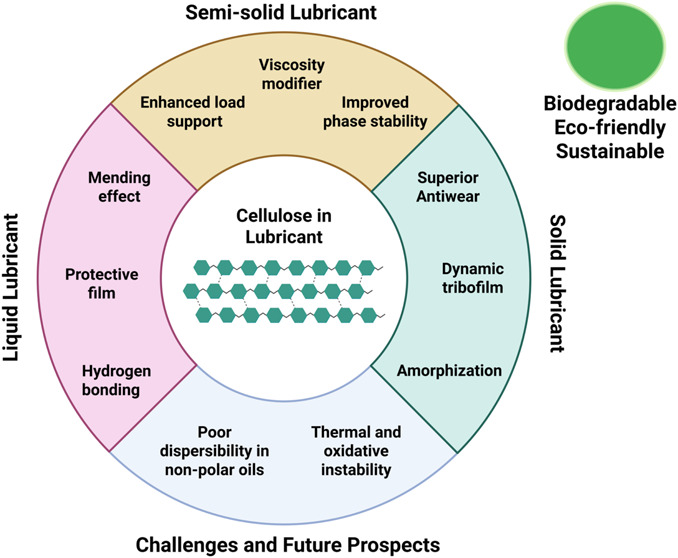

## 1 Introduction

The rapid transformation of industry driven by the Fourth Industrial Revolution and the integration of regional economic frameworks such as the ASEAN Economic Community have intensified the demand for innovative, competitive, and sustainable technological solutions.^1^ In this context, sectors such as manufacturing and mechanical systems face increasing pressure to enhance performance, reduce energy losses, and extend component life through improved friction and wear control. As a result, tribology—the science of friction, wear, and lubrication—plays a pivotal role in advancing industrial competitiveness and energy efficiency.^2^ One of the popular solutions is by adding additional additives, such as nanoparticles.

Nanoparticles, typically measuring less than 100 nm in any direction, possess a high surface area-to-volume ratio and unique nanoscale reactivity, making them attractive lubricant additives with promising tribological properties.^3–11^ For example, incorporating ZnO nanoparticles at a concentration of 0.5 wt % resulted in an approximate 8% reduction in friction coefficient and a 5% decrease in pin wear.^12^ Furthermore, the utilization of advanced nanoparticles like graphite oxide led to significant improvements, with friction and wear reductions of approximately 20 and 18%, respectively.^13^ However, despite their promising advantages, the use of nanoparticles in lubrication can present challenges related to dispersion, environmental and safety concerns, and relatively high costs.^14,15^

Cellulose, a renewable natural polymer, has emerged as a promising candidate for enhancing lubrication and grease formulations due to its eco-friendly and sustainable characteristics.^16^ Its unique combination of mechanical strength, chemical stability, and biodegradability makes cellulose an attractive choice for various industrial applications.^17–20^ Although cellulose is traditionally associated with applications in paper, textiles, and packaging, its adoption in the realm of lubrication and tribology has raised intriguing possibilities for enhancing the performance and sustainability of lubricants and greases.^21,22^

This review aims to provide a comprehensive overview of the role of cellulose-based additives in lubrication systems, focusing on their tribological performance, underlying mechanisms, and potential applications. It examines how cellulose contributes to friction and wear reduction through mechanisms such as protective film formation and surface smoothing. By synthesizing current literature, the paper highlights both the benefits, including biodegradability, chemical stability, and mechanical strength, and the limitations of incorporating cellulose into lubricants and greases. As the demand for sustainable and environmentally responsible lubricants continues to grow, understanding the capabilities and challenges of cellulose-based additives has become increasingly important for the development of efficient and durable lubrication technologies.

## 2 Lubrication Mechanism of Cellulose as Lubricant

Cellulose, when used as a lubricant, can operate in two distinct modes: as an additive to liquid or semisolid lubricants and as a solid lubricant within composites or hydrogels. In its role as a lubricant additive, cellulose is typically processed to reduce its particle size. This size reduction is essential for ensuring effective dispersion within the base lubricating fluid.^23,24^ Similar to the concept of nanofluids, nanoparticles are incorporated into base fluids to enhance their tribological properties. This method improves the stability and promotes efficient blending with the lubricant, contributing to a more sustainable lubrication approach. Alternatively, cellulose can function as a solid lubricant when embedded in composites or hydrogels.^25^ In this form, cellulose-based materials possess unique properties that reduce friction and wear between surfaces. These solid lubricants are known for their durability and reduced maintenance requirements, making them particularly advantageous in situations where traditional lubricants are not suitable or environmentally friendly.

Several mechanisms of cellulose-based lubricants or grease for friction reduction and wear have been discovered in the literature. Generally, the mechanism is similar to that of nanoparticles. However, there are certain aspects that give cellulose an advantage over nanoparticles. Its lubrication mechanisms are rooted in its chemical structure and physical characteristics. Cellulose is hydrophilic due to the abundance of hydroxyl (−OH) groups along its polymer chains. When cellulose is incorporated into lubricants or grease, it interacts with water or other polar molecules, creating a protective boundary layer that adheres to the surfaces. This layer reduces friction and wear, particularly under high-pressure or high-temperature conditions.

Li et al. incorporated cellulose nanocrystals (CNCs) into polyalphaolefin oil, with CNCs measuring 100–300 nm in length and 10–20 nm in width. Because of the small size of the CNCs, they could fill the scars or grooves on the friction surface, resulting in compensation for the mass loss.^26^ This phenomenon is known as the mending effect, which is a popular lubrication mechanism of nanoparticles.^27^ For cellulose to perform this mending effect effectively, its particle size must be reduced, which can be achieved through sonication. Sonicated cellulose nanocrystals (CNCs), precisely sized to 100–300 nm in length and 10–20 nm in width, can then disperse uniformly and effectively fill surface defects.^28^

This behavior is visually depicted in Figure 1, which illustrates the dual mechanisms of CNC-based lubrication in PAO oil: (1) mending effect, where CNCs fill worn areas to restore surface topography, and (2) chain entanglement between modified CNCs (mCNCs), C18 fatty chains, and PAO oil chains, forming a physically entangled structure that enhances load-bearing capacity and lubricant film stability during sliding. These synergistic effects contribute to reduced friction and the extended service life of lubricated components.

**Figure 1:**
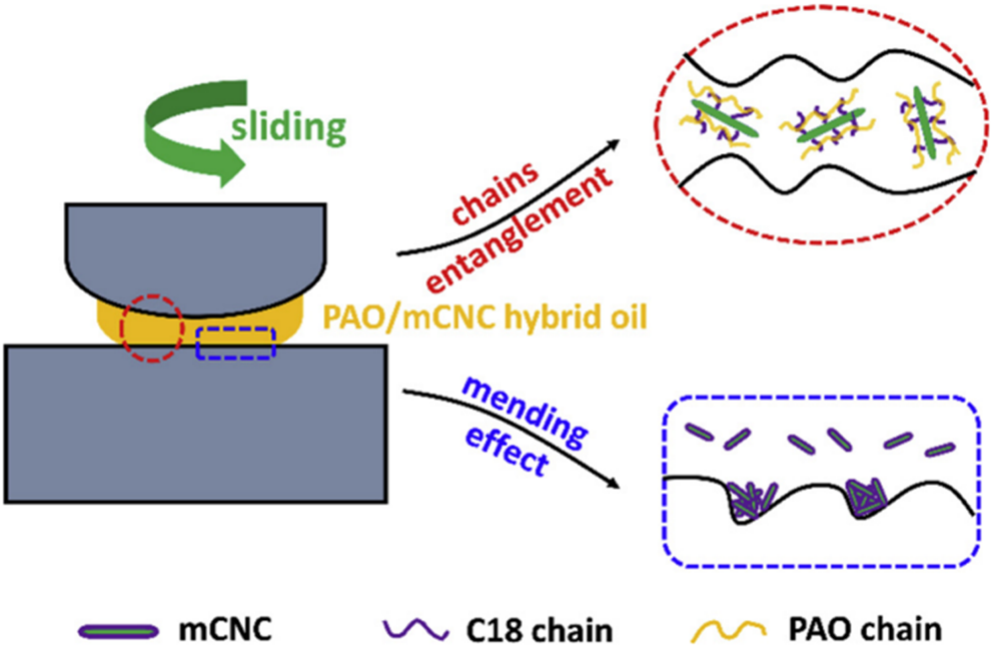
CNC lubricating mechanism in oil. Reproduced with permission from ref 26.

Another recent development has explored CNCs as a vital component in forming supramolecular hydrogel lubricants with enhanced tribological performance. Zhou et al. reported the synthesis of a bicomponent hydrogel by blending CNCs with diglycerol (DG) in deionized water (DW), forming a viscoelastic three-dimensional (3D) network via extensive hydrogen bonding.^29^ This CNC/2.4-DG/0.1 hydrogel showed superior friction reduction and wear resistance compared to individual CNC in DW. The key lubrication mechanism was attributed to a synergistic mending effect, where CNC and DG coadsorb onto sliding interfaces to repair surface defects while forming a thin protective film. Additionally, the presence of dynamic H-bond interactions between CNC and DG contributes to dissipating frictional energy, further enhancing the antiwear characteristics.

These findings are visually summarized in Figure 2, adapted from Zhou et al., which schematically illustrates the comparative mechanisms of the DW + CNC and CNC/2.4-DG/0.1 systems. In the DW+CNC case, CNCs adsorb onto tribo-interfaces, forming a thick compact film, while in the hydrogel system, DG molecules act as nanolinkers, promoting coadsorption with CNCs to form a thinner yet more efficient protective layer. The dynamic network allows better conformability and stress dissipation, especially under variable sliding conditions.

**Figure 2:**
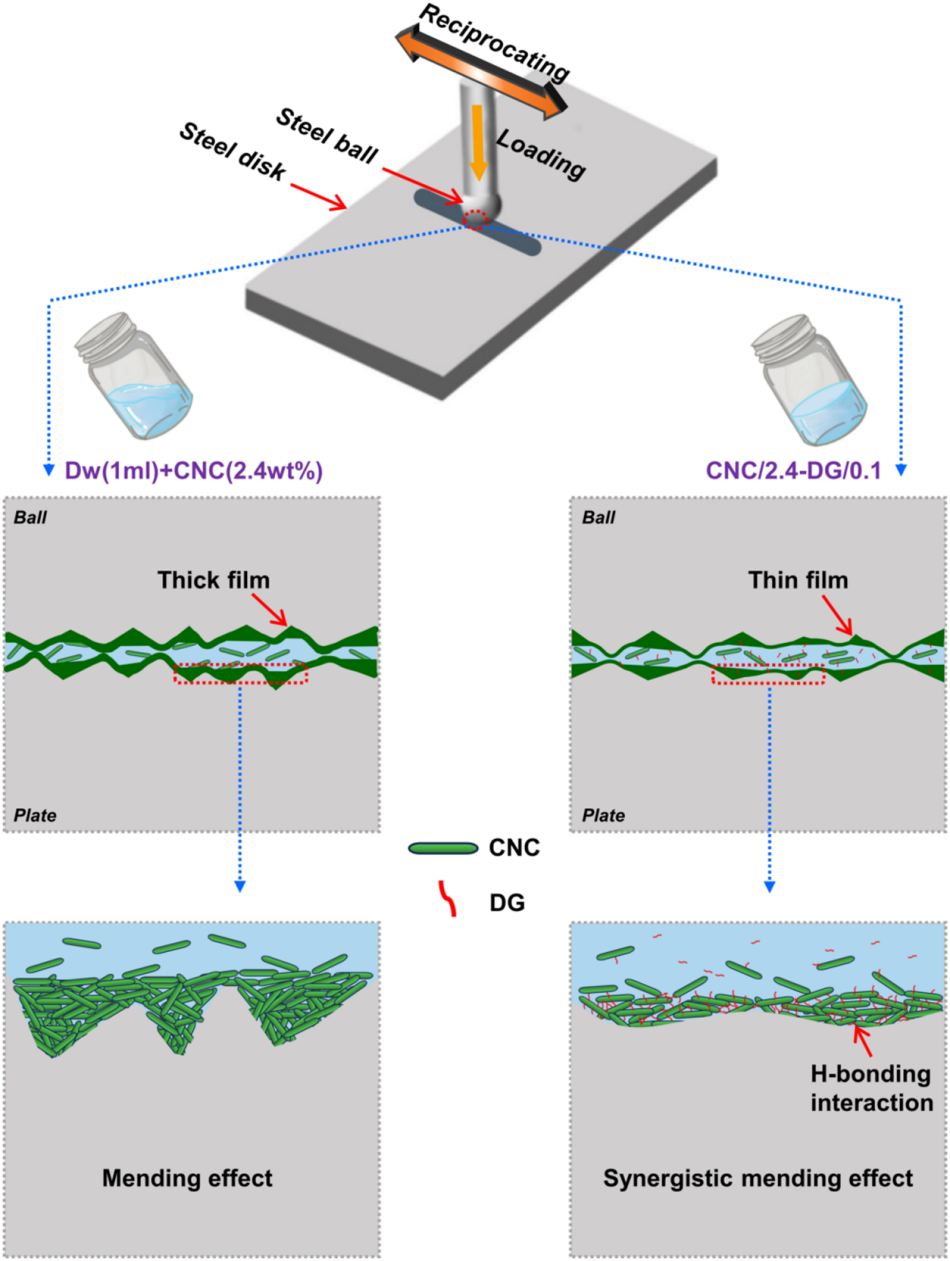
Schematic illustration of the friction reduction and antiwear mechanism of CNC in DW and CNC/2.4-DG/0.1 supramolecular hydrogel. The CNC/2.4-DG/0.1 system exhibits a synergistic mending effect and an energy-dissipating H-bonding network, resulting in improved tribological performance. Reproduced with permission from ref 29.

## 3 Cellulose as a Liquid Lubricant

Different forms of cellulose, such as CNCs, cellulose nanofibers (CNFs), and other cellulose derivatives, are increasingly used in liquid lubricants to enhance performance through their unique structures and properties. This section explores how each type functions in liquid lubricants, highlighting its specific mechanisms and benefits.

### 3.1 Nanocellulose

Nanocellulose refers to cellulose-based nanomaterials that include CNCs, cellulose nanofibers (CNFs), and bacterial cellulose (BC), each with distinct structural and functional characteristics. Cellulose nanocrystals are highly crystalline, rod-like particles with exceptional strength and stiffness.^30,31^ Cellulose nanofibers are longer, flexible fibrils with a networked structure, providing a high surface area and flexibility.^32^ Bacterial cellulose, produced by specific bacterial strains, is a highly pure form of cellulose with a unique three-dimensional structure, known for its excellent water-holding capacity and mechanical robustness.^33,34^

Various studies in recent years have explored the use of nanocellulose as eco-friendly additives in different liquid lubricants.^26,35–37^ The effect of nanocellulose addition on the friction and wear performance of several lubricants is shown in Table 1. Li et al. added CNCs at various concentrations to polyalphaolefin (PAO) oil and conducted tribological tests using ball-on-disk configurations under different operating conditions. The results indicated that the coefficient of friction (COF) decreased by up to 30% compared to base oils when CNCs were used at an optimal concentration.^26^

**Table 1:** Summary of Recent Studies Related to the Usage of Nanocellulose as a Liquid Lubricant Additive

base oil and cellulose type	details	tribological test method	key findings	refs
water + CNC pulp	cellulose pulp (Eucalyptus kraft), 96–98% purity	pin-on-disk tribometer	53% COF reduction with 1 wt % CNCs (63 rpm, 8.47 N load)	^37^
	length: 161–420 nm	load: 1.77 and 8.47 N	99% wear rate reduction with 1.25 wt % CNCs (500 rpm, 8.47 N)	
	diameter: 7–12 nm	speed: 500 and 63 rpm		
water + modified CNCs	S-CNC (CNCs with sulfonyl group) and C–CNC (CNCs with carboxyl group)	universal Mikro Tribometer	55.8% COF improvement with 0.05 wt % C–CNC	^36^
		load: 7 N	the optimum CNC concentration for COF improvement was 0.05 wt %; higher concentrations showed no significant improvement	
	particle size: S-CNC (333 nm); C–CNC (112 nm)	frequency: 4 Hz		
water + CNC powder	CNC powder (CelluForce Inc.)	pin-on-cylinder tribometer	adding 2 wt % CNCs reduced COF to 0.09, a quarter of the COF without CNCs, and decreased wear depth and width by over 50%	^44^
	particle size: 1–50 μm	load: 50 N		
		rotation speed: 130 rpm		
water + CNC powder	CNC powder (CelluForce Inc.)	pin-on-disk flat tribometer	within the optimum range of 1–2 wt % CNCs, the COF is reduced by 50% compared to water	^40^
		load: 5, 10, and 15 N		
		rotation speed: 50 rpm		
water + CNCs (sonicated)	sonicated CNCs (CelluForce Inc.)	pin-on-disk flat tribometer	sonicated CNCs improve COF and wear by 25% and 30%, respectively, compared to nonsonicated CNCs	^28^
		load: 15 and 20 N		
		rotation speed: 50 rpm		
engine oil (SAE 40) + CNCs	extracted from acetate-grade dissolving pulp from the Western Hemlock plant (Blue Goose Biorefineries Inc.)	piston skirt-liner tribometer	0.1 wt % CNCs significantly reduced COF, especially at low loads and high speeds.	^38^
	particle size: 75.3 nm	load: 39.2 and 98.1 N	0.1 wt % CNC achieved up to 69% wear reduction under high load and low speed compared to SAE 40 oil	
		rotation speed: 500 and 200 rpm		
engine oil (SAE 40) + CNCs	hybrid: CNCs and copper(II) oxide (CuO) particles	custom-designed tribometer with piston ring–cylinder configuration	the 0.5 wt % CNC-CuO nanolubricant reduced COF by 48–50% in boundary lubrication and improved wear rate by 33.5%, lowering scuffing and microabrasive wear compared to SAE 40	^45^
		load: 40 and 100 N		
		rotation speed: 200–500 rpm		
engine oil (SAE 40) + CNCs	hybrid: CNCs and copper(II) oxide (CuO)	custom friction and wear tester with a reciprocating motion	at 0.3% concentration, CNC-CuO reduced COF by 30.95% in high-speed, low-load conditions and improved performance in boundary lubrication.	^46^
	particle size: 82.6 nm	low and high load		
PAO oil + CNCs	CNCs were isolated from native cotton via hydrochloric acid hydrolysis.	ball-on-disk tribometer	at 2 wt %, mCNC in PAO oil reduced COF by 30% and decreased wear depth, achieving a smoother wear track than CNCs or pure PAO.	^26^
	CNCs were modified with stearoyl chains (mCNC) for improved compatibility with PAO oil.	load: 60 N		
	length: 100–300 nm	rotation speed: 20 rpm		
	diameter: 10–20 nm			
engine oil (10W30) + CNF	lab-prepared CNF from rice straw	block-on-ring tribometer	the optimal CNF concentration (2 wt %) led to a 31% reduction in wear scar width compared to the unmodified spent oil sample	^41^
	particle size: 30.53 ± 17.6 μm^2^	load: 200 N	CNF improved the viscosity, lubrication, and antiwear performance of the oil and reduced friction, vibration, and noise.	
	nanosized fraction: 67.57 ± 5.5%	rotation speed: 500–2500 rpm		
polyol ester (POE) oil + BC	TEMPO-oxidized BC powder	ball-on-disk tribometer	the addition of TEMPO-oxidized BC into POE oil resulted in a reduced COF by up to 40%.	^42^
	BC was prepared from a *Komagataeibacter xylinu*s pellicle	load: 5.5 N	wear rate reduction by about 49%, attributed to the formation of a smoother wear track and reduced contact area on the sliding surfaces.	
	particle size: ± 240 μm	rotation speed: 200 rpm		
	diameter: < 100 nm			
polyol ester (POE) oil + BC	hybrid: TEMPO-oxidized BC and graphene nanoparticle.	ball-on-disk tribometer	the combination of graphene and BC reduced the CoF by up to 73% compared to the base POE lubricant.	^43^
		load: 10 N	tests conducted at 65 °C showed that the BC-based additive maintained its effectiveness at elevated temperatures.	
		frequency: 10 Hz		
		temperature: 65 ± 2 °C		

Furthermore, CNCs with sulfonyl group addition (CNC-S) at 0.5 wt % in water reduced the COF by nearly 63%. This enhancement is due to the adsorption of polar groups like hydroxyl and carboxyl onto the surface, forming a protective film, while the liquid crystalline structure of S-CNC aids in creating an ordered lubricating layer, improving frictional properties.^36^ Awang et al. conducted a study of the tribological properties of CNCs as a lubricating additive in engine oil. Using a piston skirt-liner tribometer, they tested various CNC concentrations in SAE 40 oil and observed that only 0.1 wt % CNC provided the best results, reducing the wear rate by up to 69% under high-load, low-speed conditions.^38^

CNCs are especially popular in water-based lubricants due to their natural hydrophilicity and ease of dispersion in aqueous solutions.^39^ When used at 1 wt % concentration in water, CNCs have been shown to reduce the CoF by approximately 50% compared to water alone.^40^ In another study, Da Rocha et al. added CNCs to water-based lubricants, which significantly reduced wear rates under severe testing conditions (500 rpm, 8.47 N), achieving up to a 99% reduction compared to the control. This substantial improvement is attributed to CNCs’ ability to form a protective film over the contact surface and polish the disk, minimizing material removal and smoothing the surface.^37^

Similar to CNCs, CNFs have also gained attention as eco-friendly additives in lubricants due to their unique fibrous structure, which provides a high surface area and enhances interaction with lubricant bases. Studies have demonstrated that CNF, when added to lubricants, can significantly improve both the friction and wear properties. For instance, Taha et al. investigated the addition of CNF to spent engine oil, revealing significant enhancements in viscosity and antiwear properties. At a concentration of 2.0 wt %, CNF increased the kinematic viscosity by 14.5%, resulting in a more stable lubricant film under operational stress. Additionally, the presence of CNF reduced the wear scar width by 31% compared to untreated oil, demonstrating its ability to improve the longevity and durability of engine parts by minimizing direct metal-to-metal contact and wear.^41^

Moreover, BC has also shown promise as an eco-friendly additive in lubricants. Our previous studies have demonstrated that TEMPO-oxidized BC, when incorporated into polyol ester (POE) oil-based biolubricants, enhances viscosity and tribological performance. The TEMPO oxidation process plays a key role in facilitating the separation of BC into individualized nanofibers. This individualization increases the surface area of BC, enabling better dispersion within the lubricant and enhancing interaction with contact surfaces. TEMPO-treated BC in POE oil can significantly reduce the COF and wear rate, creating a smoother contact interface and reducing wear scars by up to 49%.^42^

Another study by Fuadi et al. reported that the addition of BC, particularly when combined with graphene nanoplatelets, further enhances the lubricant’s tribological properties, reducing the specific wear rate by 20% and lowering friction under high-load, low-speed conditions. This improvement is attributed to the individualized BC fibers’ ability to form a protective tribofilm on sliding surfaces, minimizing material removal and wear. The individualized fibers offer a continuous and even tribofilm, which effectively reduces friction and protects surfaces in demanding lubrication environments.^43^

### 3.2 Modified or Functionalized Cellulose

Modified or functionalized cellulose has emerged as a promising additive in liquid lubricants due to its tailored surface properties, which enhance compatibility and performance in diverse lubrication systems. Among these, carboxymethyl cellulose (CMC) and cellulose acetate stand out for their effectiveness in water- and oil-based lubricants, respectively, offering distinct benefits that meet specific lubrication needs. CMC is particularly suited to water-based lubricants, where its water solubility and hydrophilic carboxymethyl groups enable it to form a stable, hydrated layer on contact surfaces.^47^ This layer reduces the COF and wear by minimizing direct metal-to-metal contact.

Additionally, CMC acts as an effective surfactant, providing electrostatic stabilization to disperse nanoparticles within lubricant formulations. Our previous studies have shown that CMC can stabilize nanoparticles in fluid, preventing aggregation through electrostatic repulsion.^47^ In particular, CMC achieved significant performance as an additive in liquid lubricant. The MXene/CMC composite with 0.4 wt % CMC in water demonstrated a 25% reduction in COF compared to water alone, indicating a stable lubricating layer with minimal nanoparticle aggregation.^48^ Additionally, the CMC/MXene composite in crude palm oil (CPO) reduced the COF by 49% and wear rate by 74% under high-load, high-speed conditions, achieving superior wear protection.^49^

Cellulose Acetate is more suitable for oil-based liquid lubricants due to its esterified structure, which improves its compatibility with nonpolar lubricant bases. Unlike CMC, cellulose acetate is not water-soluble but acts as a robust thickening agent in oil-based systems. This thickening effect stabilizes the viscosity of the lubricant at elevated temperatures, preventing thinning under high shear and maintaining lubricant effectiveness in high-stress environments. The thermal stability of cellulose acetate also makes it ideal for applications exposed to sustained high temperatures, such as in industrial machinery or automotive engines. In comparison with nanocellulose (CNC, CNF), which can reinforce oil-based lubricants, cellulose acetate provides more consistent thickening and thermal resilience, ensuring long-lasting protection and viscosity stability across a range of operating conditions.

While nanocellulose forms like CNCs and CNFs are valuable in both water- and oil-based lubricants due to their ability to form protective nanofilms, CMC and cellulose acetate offer easier integration and stability in their respective bases. CMC, with its surfactant properties and electrostatic stabilization, supports both nanoparticle dispersion and friction reduction in water-based systems. Cellulose acetate delivers thermal resilience and viscosity control in oil-based formulations. These properties make modified celluloses such as CMC and cellulose acetate versatile and efficient additives for high-performance, sustainable liquid lubricants, addressing the specific challenges of different lubrication environments while complementing the unique properties of nanocellulose-based additives. The effects of modified or functionalized cellulose additives on the friction and wear performance of various lubricants are shown in Table 2.

**Table 2:** Summary of Recent Studies Related to the Use of Modified or Functionalized Cellulose as a Liquid Lubricant Additive

base oil and cellulose type	details	tribological test method	key findings	refs
water + CMC	CMC powder was purchased from Showa Chemical Co., Ltd.	ball-on-disk tribometer	the addition of CMC improved the coefficient of friction (COF) and wear rate, decreasing them by approximately 19.7% and 59%, respectively, compared to pure water.	^47^
	pH: 6.0–8.0	load: 16 N	CMC significantly enhanced the stability of the nanofluid, with zeta potential values reaching –72.29 mV, indicating high dispersion stability.	
	additional additives: Tannic acid and Al_2_O_3_ nanoparticles	rotation speed: 191 rpm		
water + CMC	CMC powder (Changsu Wealthy Science and Technology)	pin-on-disk tribometer	the addition of CMC achieved a COF of 0.169, which is significantly lower than that of water.	^50^
	additional additives: Uncaria gambir powder	load: 11.76 N		
		sliding speed: 0.2 m/s		
water + CMC	CMC powder (Changsu wealthy science and technology)	pin-on-disk tribometer	the sample with 0.35 wt % MXene and 0.4 wt % CMC achieved an average COF of 0.26, a 56% reduction compared to water alone (4 h test).	^44^
		load: 10 N		
	additional additives: MXene nanoparticle	duration: 4 h	the presence of CMC improved stability and created a uniform lubricating layer, which effectively minimized wear and surface corrosion.	
water + Methylcellulose (MC)	MC was produced by Yuan Ye company	HSR-2 M friction and wear tester (ball-on-disk)	the addition of 0.6 wt % MC reduced the COF by 59% and the wear rate by 40% compared to pure water.	^51^
	additional additives: Gallium-based liquid-metal nanoparticles	load: 10 N Duration: 20 min		
water + hydroxyethyl cellulose (HEC)	HEC was purchased from Sigma-Aldrich	ball-on-disk tribometer	the addition of 0.5 wt % ZnO + 0.6 wt % HEC led to an 84.6% reduction in COF, compared to pure water lubrication.	^52^
	additional additives: ZnO nanoparticles	load: 5–30 N Duration: 30 min	the lubricant formed a hydrated molecular layer, preventing adhesion and improving tribological performance.	
			HEC enhanced dispersion, preventing ZnO nanoparticle aggregation and improving film stability.	
castor oil + cellulose acetate (CA)	CA was purchased from Sigma-Aldrich	ball-on-plate tribometer	3 wt % CA dispersions exhibited the best lubrication performance.	^53^
		load: 10, 20, and 40 N	CA fibers formed a protective lubricating layer, minimizing wear and improving oil retention.	
		sliding speed: 4 0.05 to 1400 mm/s		

### 3.3 Other Cellulose-Based Materials

In addition to nanocellulose and conventional cellulose derivatives, a wide range of alternative cellulose-based materials, particularly cellulose esters and hybrid composites, have gained attention for their application in green lubrication systems. These materials offer surface-active functionalities, thermal and oxidative stability, and structural tunability that make them attractive for various tribological environments.

Among the earliest examples, cellulose dodecenylsuccinate ester (CDDS), synthesized via esterification with dodecenylsuccinic anhydride, was reported by Singh et al. to significantly reduce friction and wear when blended with *n*-decane.^54^ A higher degree of substitution (DS) enhanced the performance, with DS = 2.03 resulting in over 25% wear reduction and a substantial drop in the friction coefficient. The amphiphilic structure of the ester enabled strong adsorption of unmodified hydroxyl groups onto metal surfaces, while the hydrophobic chains provided a low-shear interface to mitigate friction.

Zhang et al. further demonstrated the potential of nanocellulose fatty acid esters (NC-FAEs), such as stearate-, laurate-, and oleate-modified cellulose.^55^ These esters, used at 1 wt % in paraffin oil, exhibited excellent tribological behavior, particularly under boundary lubrication conditions. The NC-stearate showed the most pronounced effects due to its ability to form dense, stable boundary films that inhibited metal-to-metal contact and wear propagation.

More recently, Yang et al. reported the in situ synthesis of a cellulose acetate-laurate-based nanocomposite containing calcium borate.^400^ This hybrid material functioned as an efficient extreme pressure (EP) and antiwear additive in liquid paraffin. The composite structure offered excellent thermal decomposition resistance and exceptional tribological stability. At 2 wt % loading, the composite reduced wear scar diameter from 694 μm (for pure oil) to 311 μm and achieved a 4-fold reduction in friction coefficient under EP conditions. The synergistic combination of the laurate chain, cellulose acetate backbone, and calcium borate nanoparticles facilitated strong surface adsorption, energy dissipation during asperity interactions, and a tribochemically stable protective layer.

The lubrication mechanism of acid esterified cellulose nanocrystal (AEC) additives has also been illustrated in recent studies.^56^ As shown in Figure 3, the additive is capable of forming multiple protective layers on the sliding interface, including adsorption films via ester group interactions, chemical transfer films through tribochemical reactions with metal surfaces, and entanglement cross-link networks that retain oil molecules within nanosized pores. These synergistic mechanisms contribute to the formation of stable low-friction, low-wear conditions even under mechanical and thermal stress.

**Figure 3:**
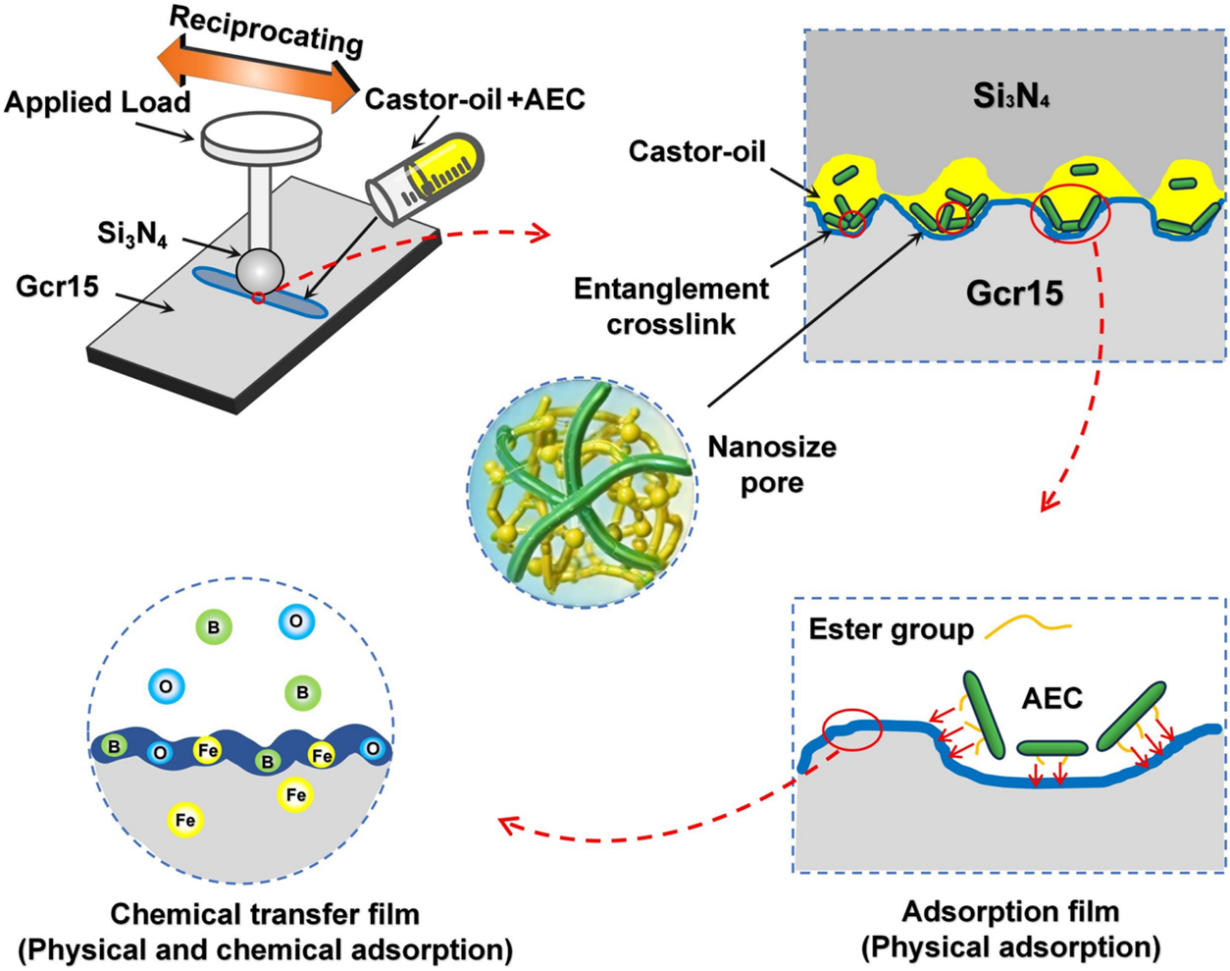
Schematic illustration of the lubrication mechanism AEC nanoadditive in castor oil. Reproduced with permission from ref 56.

## 4 Cellulose in Semisolid Lubricants

Semisolid lubricants, commonly known as greases, consist of a thickening agent dispersed in a lubricating fluid. Cellulose and its derivatives have emerged as promising biobased thickeners due to their ability to form a stable three-dimensional network that entraps oil. For instance, Shetty et al.^21^ reported the development of PEG/water-based lubricating gels incorporating two cellulose derivatives: hydroxyethyl cellulose (HEC) and sodium carboxymethyl cellulose (NaCMC).

The gels exhibited semisolid rheology and effective antiwear performance. The lowest wear values were achieved with 1–1.25% HEC and 1% NaCMC, with NaCMC gels consistently outperforming HEC in both disc and ball wear. This was attributed to NaCMC’s anionic nature, which enhanced its adsorption to metal surfaces. Furthermore, the authors proposed that the hydrated polymer layer formation and shear-induced alignment of the cellulose chains helped stabilize the lubricating film and reduce asperity contact under sliding conditions.

Expanding on this, Ilyin et al.^57^ developed biodegradable greases using unmodified microfibrillated cellulose (MFC) in triethyl citrate. These MFC-based greases exhibited shear-thinning behavior, viscoelasticity, and yield stress typical of conventional greases. At low concentrations (1–3%), MFC reduced wear by filling in surface irregularities. At higher loadings (5–7%), the formation of a smoother nanocellulose-rich tribofilm further lowered friction, decreasing the steady-state friction coefficient to 0.071–0.077, markedly below that of the base oil without MFC (∼0.1).

Further insights were provided by a recent study,^58^ which investigated CNC as nanoadditives in lithium-based greases of varying consistency. CNC was found to reduce friction and wear through a combination of surface mending, tribofilm formation, and load-bearing reinforcement. CNC’s high mechanical strength and nanoscale size enabled effective migration to the contact zone, outperforming conventional additives like CaCO_3_ in both wear and friction reduction.

The tribological mechanisms of cellulose in semisolid lubricants align closely with those observed in liquid lubricants. Core effects, such as the mending effect, formation of protective tribofilms, and viscosity modification, remain central to performance enhancement in both systems. However, greases offer a distinct structural environment wherein cellulose not only modifies viscosity but also acts as a three-dimensional scaffold, creating a physically entangled network that traps oil and resists phase separation under stress.^59^

This structural thickening capability is a unique advantage in greases, enabling the maintenance of lubricant consistency and film stability even under prolonged shear or load conditions, where liquid lubricants may fail. Moreover, the ability of cellulose to simultaneously reinforce the grease matrix and modulate tribological interfaces underscores its multifunctional role, making it a compelling candidate for biobased, high-performance grease formulations.

## 5 Cellulose in Solid Lubricant

Solid lubricants are crucial in extreme conditions where conventional lubricants (liquid or semisolid) fail due to high temperature, vacuum, or exposure to radiation. Cellulose, in its various forms and composites, has recently garnered attention for application in solid lubrication systems owing to its renewable nature, chemical tunability, and tribological performance. Cellulose can be engineered into solid lubricants by direct compaction or by blending with other materials to form composite structures.^60^

One example is the work by Shi et al., who explored hydroxypropyl methylcellulose (HPMC) composite coatings reinforced with nanosized metals (Al, Cu) and metal oxides (Al_2_O_3_, CuO) to improve tribological performance under dry-sliding conditions.^61^ Their findings revealed that metal additives such as Cu, when embedded in the cellulose matrix, accumulated at the wear interface and ruptured under load, forming a third-body tribofilm layer. This behavior is categorized under the S3M2 mode in the third-body theory, where the tribofilm mediates load and velocity through rupture mechanisms. In contrast, metal oxide nanoparticles (Al_2_O_3_ and CuO) followed an S3M4 mechanism, wherein expelled nanoparticles rolled within the wear track, offering superior lubrication through rolling velocity accommodation. Notably, a 2 wt % CuO additive achieved minimal wear and the lowest coefficient of friction, making it the most effective composition. These results reinforce the potential of cellulose-matrix-based composites, especially when functionalized with suitable inorganic fillers, for solid-state lubrication applications.

Another notable study by Lin et al. investigated the synergistic effects of CNCs and graphene as hybrid fillers in styrene–butadiene rubber/natural rubber (SBR/NR) composites for solid lubrication applications.^62^ The inclusion of CNCs significantly improved the friction and wear behavior of the rubber matrix due to their high mechanical strength and strong interfacial bonding with the polymer. When used in combination with graphene, the hybrid fillers provided a dual mechanism: graphene acted as a solid lubricant by forming a low-shear tribofilm, while CNCs enhanced the load transfer and matrix reinforcement. The optimized CNC/graphene hybrid formulation exhibited a 42.3% reduction in the friction coefficient and over 50% reduction in wear rate compared to neat SBR/NR. These results demonstrate the viability of cellulose-derived nanomaterials as performance-enhancing additives in flexible, solid–lubricant matrices, particularly when strategically combined with two-dimensional (2D) materials like graphene.

Furthermore, cellulose nanofibers have been compacted into dense, moldable solids to be used directly in dry-sliding applications. Okubo et al. reported the fabrication of molded 100% CNF specimens and evaluated their tribological performance under dry and boundary lubrication conditions against steel surfaces.^63^ Their results revealed that molded CNF exhibited low and stable friction coefficients (∼0.1 to 0.15) and excellent wear resistance under both lubrication regimes. Notably, wear debris formed during sliding served as a secondary lubricating layer, contributing to a prolonged tribological performance. The study also demonstrated that surface densification and controlled molding conditions critically influence the tribological characteristics.

A notable example is illustrated in Figure 4, which depicts the friction and wear mechanisms of CNF/steel tribopairs under dry-sliding conditions. According to Okubo et al., tribochemical reactions between iron and cellulose occur during sliding facilitated by localized friction-induced temperature rises. These interactions, coupled with mechanical stress, promote partial amorphization of the initially crystalline CNF in the contact region. The transition from a crystalline to an amorphous phase results in a soft interfacial layer with reduced stiffness. This localized reduction in stiffness contributes to smoother sliding and acts as a sacrificial film that absorbs energy and mitigates surface wear. As the sliding progresses, this amorphous layer continues to evolve and serves as a dynamic tribofilm, effectively delaying wear progression and enhancing the service life of the CNF-based solid lubricant system.

**Figure 4:**
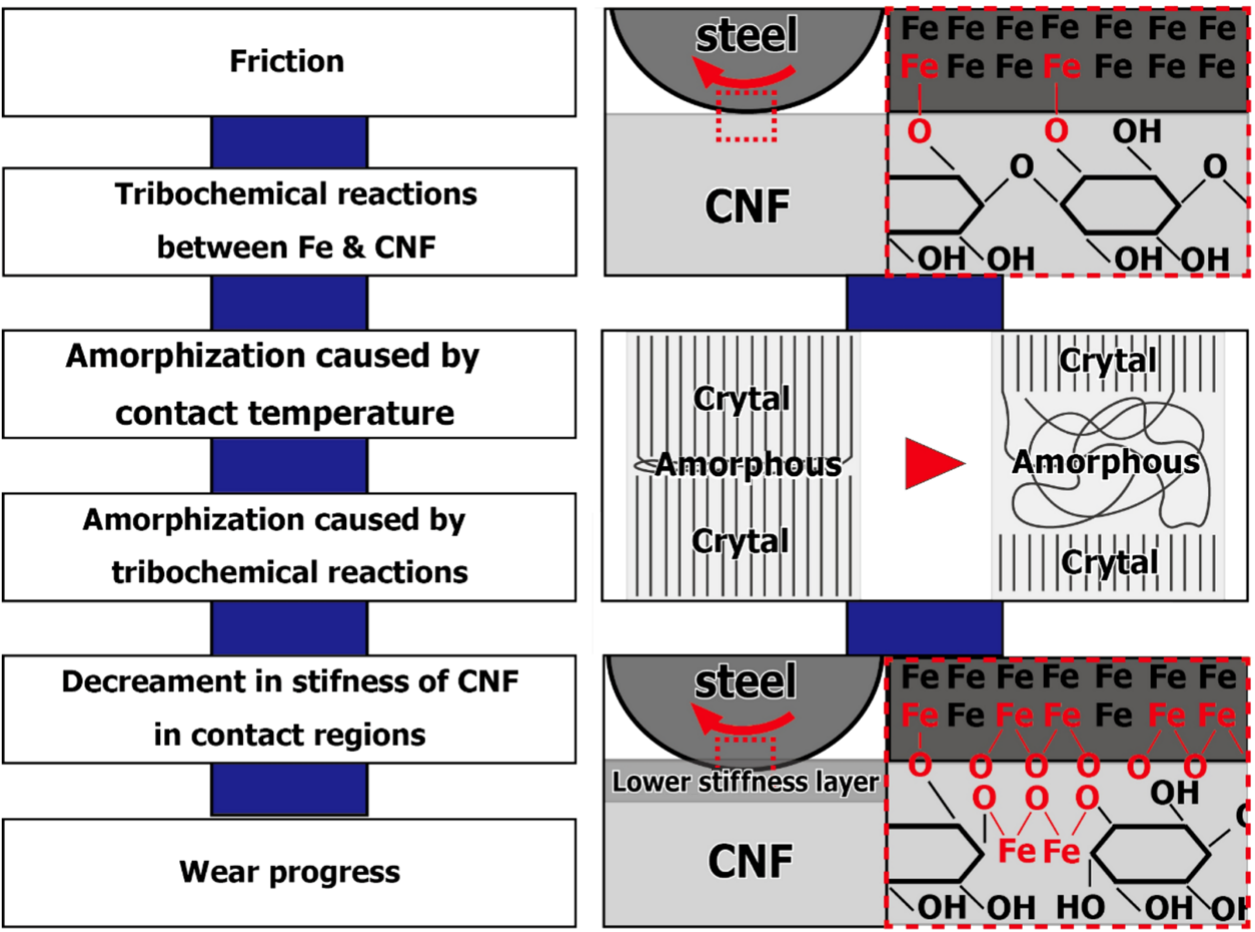
Schematic of the cellulose-based solid lubricant mechanism. Reproduced from ref 63, which was published under the Creative Commons Attribution 4.0 International License (http://creativecommons.org/licenses/by/4.0/).

A recent study by Shi et al. provided further insight into the utility of nanocellulose in thermoplastic matrices.^64^ In this work, CNCs and CNFs extracted from rice stalks were incorporated into poly­(methyl methacrylate) (PMMA) at weight ratios of 0.1–1.0 wt %. The resulting composites exhibited significantly enhanced tribological performance, with wear volume reduced by 72–90% compared to neat PMMA. The reduction in wear and friction was attributed to the formation of a durable tribolayer and mechanical reinforcement of the PMMA matrix. CNF-reinforced composites consistently outperformed CNC-based ones owing to their higher aspect ratio and ability to entangle more effectively with the polymer chains, thus improving structural integrity. This behavior is clearly illustrated in Figure 5, where both the wear volume and average coefficient of friction are shown to decrease markedly with increasing nanocellulose content, especially for CNF-reinforced PMMA.

**Figure 5:**
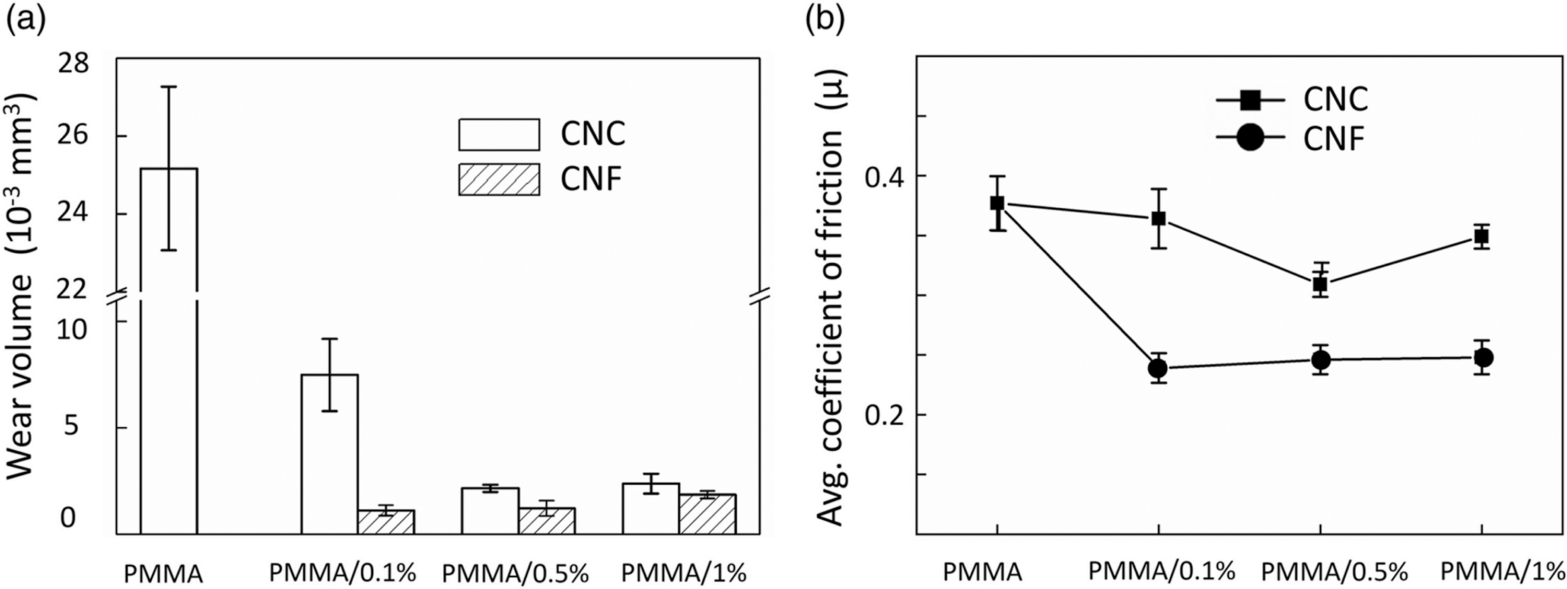
Tribological properties of nanocellulose/PMMA composites: (a) wear volume and (b) average coefficient of friction. Reproduced from ref 64, which was published under the Creative Commons Attribution 4.0 International License (http://creativecommons.org/licenses/by/4.0/).

## 6 Challenges and Future Prospects

### 5.1 Challenges

A major barrier to the effectiveness of cellulose-based lubricants lies in the poor dispersion of cellulose, particularly nanocellulose, within nonpolar lubricant matrices such as synthetic oils and greases. The intrinsic hydrophilicity of cellulose leads to agglomeration in oil-based systems, diminishing its tribological performance.^65^ Though surface modifications, such as esterification, grafting, and surfactant-assisted dispersion, have shown promise, they may involve environmentally unfriendly chemicals or reduce biodegradability. Future work must focus on developing green, cost-effective surface treatments that maintain the eco-friendly nature of cellulose while improving its dispersion and compatibility in diverse lubricant systems.

Cellulose tends to degrade or lose effectiveness under elevated temperatures, oxidative conditions, or high mechanical stresses.^66^ This restricts its application in demanding systems, such as internal combustion engines or high-speed bearings. Its poor thermal and oxidative stability remains a concern. Research on composite or hybrid lubricants combining cellulose with thermally stable additives (e.g., MoS_2_, graphene) may overcome this limitation, but a deep understanding of synergistic effects is still lacking.

Although cellulose is abundant, high-purity CNCs and CNFs often require complex, energy-intensive extraction processes. These processes hinder large-scale deployment and raise the cost of biolubricants. Future efforts should prioritize low-energy, solvent-free extraction methods or valorization of agricultural waste to produce cost-effective nanocellulose at scale.

The push for sustainable lubricants will inevitably require validation through environmental, toxicological, and lifecycle studies. Currently, few comprehensive LCAs or ecotoxicity profiles are available for cellulose-based lubricants. Without clear environmental credentials, regulatory approval, and consumer trust may be difficult to achieve. Future studies must systematically evaluate the biodegradability, aquatic safety, and end-of-life impacts of these lubricants.

### 5.2 Future Prospects

Despite these challenges, the future for cellulose-based lubricants remains highly promising. Research trends are pointing toward a new generation of smart, multifunctional biolubricants with the following opportunities: functionalizing cellulose with stimuli-responsive moieties (e.g., thermoresponsive and pH-sensitive) could lead to adaptive lubrication systems that self-adjust under changing operating conditions. This is particularly relevant for biomedical devices or intelligent manufacturing systems.

Combining cellulose with graphene, MXene, or metal oxides could result in hybrid lubricants that offer superior friction reduction, wear resistance, and load-bearing capacity. Such hybrids can overcome the limitations of single-material systems while maintaining their biodegradability. Beyond liquid lubrication, cellulose shows a strong potential in solid-state lubricants, coatings, and polymer composites for wear protection in structural applications. Its ability to act as both a binder and a lubricant is a significant advantage in dry or boundary-lubricated systems.

With the increasing interest in circular economy models, cellulose derived from agricultural byproducts (e.g., rice straw, banana fibers, ginger residues) can serve as a high-value additive in lubricant formulations. This aligns with global sustainability goals and the SDGs. For cellulose-based lubricants to enter mainstream markets, pilot-scale production, cost modeling, and industrial testing must be pursued. Partnerships among academia, lubricant manufacturers, and end-users will be essential in bridging the lab-to-market gap.

## 7 Conclusions

This review has provided a comprehensive synthesis of recent advancements in the application of cellulose and its derivatives as sustainable additives in lubrication systems, including liquid lubricants, semisolid lubricants, and solid-state formulations. The tribological benefits of cellulose materials are largely governed by their structural characteristics, chemical functionality, and interaction with the surrounding lubricant matrix, which differ in form and application context.

In liquid lubricants, CNCs primarily reduce friction and wear through the mending effect, protective boundary film formation, and chain entanglement when modified. Their high surface area and dispersibility in polar fluids facilitate adsorption onto sliding surfaces. CNFs enhance viscosity and shear resistance via network entanglement, contributing to improved load-bearing properties. BC, particularly when TEMPO-oxidized, supports film formation and smooth surface interactions, especially in polyol ester oils.

In semisolid lubricants, such as greases, cellulose serves a dual function as a rheological thickener and a tribological enhancer. It forms physically entangled networks that trap base oils and resist phase separation. These networks maintain the lubricant structure under shear and enhance wear protection via hydrated layer formation and shear-induced alignment. In MFC- and CNC-based greases, tribofilm formation, surface mending, and load-bearing reinforcement were key to reducing friction and wear, sometimes outperforming conventional additives. These effects illustrate the unique structural role of cellulose in stabilizing grease matrices while simultaneously improving the tribological performance.

In solid lubrication systems, cellulose acts both as a lubricating component and as a composite matrix. When combined with reinforcing fillers such as graphene or metal oxides, cellulose-based solid composites exhibit mechanisms, including third-body tribofilm formation, rolling accommodation, and tribochemical transformations. These mechanisms result in substantial reductions in friction and wear under dry or boundary-lubricated conditions.

Functionalized celluloses, such as CMC, HEC, and cellulose acetate, exhibit improved dispersion and film-forming capabilities. CMC provides electrostatic stabilization and effective interaction in water-based systems, while cellulose acetate offers thermal stability and viscosity control in oil-based lubricants. Their ability to stabilize nanoparticles and prevent aggregation contributes to the long-term lubricant stability and performance.

Despite these promising outcomes, several limitations persist. These include poor dispersion in nonpolar media, thermal degradation under extreme conditions, high production costs of nanocellulose, and a lack of comprehensive lifecycle and toxicity evaluations. Addressing these challenges through green functionalization, hybrid additive strategies, and scalable extraction methods is essential for industrial adoption.

Overall, cellulose-based materials exhibit a wide range of tribological mechanisms depending on their form and formulation context, making them versatile candidates for sustainable lubrication. Continued interdisciplinary research, particularly on mechanism–structure–performance relationships and industrial-scale validation, will be crucial for accelerating their transition into mainstream tribological applications.
